# Genome-wide identification and expression analysis of the *Dof* gene family under drought stress in tea (*Camellia sinensis*)

**DOI:** 10.7717/peerj.9269

**Published:** 2020-06-10

**Authors:** Qian Yu, Chen Li, Jiucheng Zhang, Yueyue Tian, Hanyue Wang, Yue Zhang, Zhengqun Zhang, Qinzeng Xiang, Xiaoyang Han, Lixia Zhang

**Affiliations:** 1 College of Horticulture Science and Engineering, Shandong Agricultural University, Tai’an, China; 2 State Key Laboratory of Crop Biology, Shandong Agricultural University, Tai’an, China

**Keywords:** Drought stress, Dof transcription factors, Gene expression, Tea

## Abstract

**Background:**

DNA-binding one zinc finger (Dof) proteins are plant-specific transcription factors important for seed development, hormone regulation, and defense against abiotic stress. Although drought stress is a key determinant of plant physiology and metabolic homeostasis, the role of *Dof* genes in different degrees of PEG6000-induced drought stress has received little attention.

**Methods:**

Tea plants (*Camellia sinensis*) were exposed to mild, moderate and severe drought stress. The Tea Genome and Plant TFDB databases were used to identify *Dof* gene family members in the tea plant. Clustal W2.1, MEGA6.0, ScanProsite, SMART, ExPASy, GSDS, MEME and STRING were used to build a phylogenetic tree, predict the molecular masses and isoelectric points of the Dof proteins, and construct a predicted protein-protein interaction network between the CsDof TFs and proteins in the *A. thaliana* database. The expression patterns of *Dof* genes in different tissues were analyzed, and qRT-PCR was used to measure the expression of *Dof* genes under different degrees of drought stress in tea.

**Results:**

We identified 16* Dof* genes in tea (*C. sinensis* cv. Huangjinya) using whole-genome analysis. Through comparative analysis of tea and* Arabidopsis thaliana*, we divided the *Dof* genes into four families (A, B, C, and D). We identified 15 motifs in the amino acid sequences of the CsDof proteins. Gene sequences and motif structures were highly conserved among families, especially in the B1 and C2 subfamilies. The protein-protein interaction network indicated that multiple CsDof proteins may be involved in the response to drought stress. Real-time PCR was used to examine the tissue-specific expression patterns of the *CsDof* genes and to measure their responses to different levels of PEG6000-induced drought stress in mature leaves. Most *CsDof* genes responded to drought stress. These results provide information on the *Dof* gene family in tea, offer new insights into the function of *CsDof* genes in a perennial species, and lay the foundation for further analysis of their functions.

## Introduction

Drought stress refers to the phenomenon of water imbalance that occurs when the plant’s water transpiration is greater than its water absorption (Wang et al., 2016). Drought seriously endangers the growth and development of crops and is one of the main environmental factors that limit plant growth and reduce crop yields (Sharma et al., 2017; Zhu et al., 2018). Under severe drought stress, the stomatal conductance, net photosynthetic rate, and transpiration rate of *Camellia sinensis* cv. Tieguanyin decreased significantly (Lin, 1998). Studies have also shown that the superoxide dismutase activity of tea increases under short-term or mild drought stress but decreases under long-term or severe drought stress, causing tea plants to age more rapidly (Liu, 2006; Fu, 2018). It is therefore important to study and improve plant drought resistance.

Transcription factors play a particularly important role in plant growth and development (Lu et al., 2009). DNA-binding one zinc finger (Dof) proteins are a subfamily of the zinc finger protein family (Wei et al., 2018a; Wei et al., 2018b). This family is unique to plants and has not been found in yeast or nematodes (Gupta et al., 2015). The common ancestor of the Dof transcription factor family was discovered in *Chlamydomonas reinhardtii*, which has only one Dof transcription factor (Moreno-Risueno et al., 2007). Dof proteins consist of a conserved N-terminal single zinc finger DNA-binding domain (the Dof domain) and a C-terminal domain (Kushwaha et al., 2011; Rueda-Romero et al., 2012; Ma et al., 2015; Chen et al., 2017). Studies have found that Dof proteins are approximately 200–400 amino acids in length (Yanagisawa & Schmidt, 1999). The Dof domain consists of 50–52 amino acids and has a classical four-cysteine zinc finger that specifically binds to the core sequence (A/T)AAAG of target gene promoters (Yanagisawa & Schmidt, 1999). This bifunctional domain mediates both DNA–protein and protein–protein interactions (Yanagisawa, 1997; Krohn, 2002). For example, the Dof transcription factor OBP1 (OBF binding protein) interacts with the bZIP transcription factors OBF4 and OBF5 (two *ocs* element binding factors) (Zhang et al., 1996). The Dof domain is thought to interact with different regulatory proteins, leading to a diversity of Dof protein functions (Noguero et al., 2013), including plant defense (Chen, Chao & Singh, 1996; Ward et al., 2005; Rueda-Romero et al., 2012), abiotic stress response (Iwamoto, Higo & Takano, 2009), auxin response (Chen & Cao, 2015), and photoperiod response (Gualberti et al., 2002; Wang et al., 2007).

Corrales et al. (2014) found that the overexpression of *SlCDF1* and *SlCDF3* (two tomato Cycling Dof Factors) significantly enhanced the drought resistance and salt tolerance of Arabidopsis and activated other stress-responsive genes such as *COR15*, *RD29A*, and *ERD10*. Complementing the work of Li et al. (2016), we have identified 16 new *CsDof* genes, thereby expanding our understanding of drought stress response in tea.

## Materials & Methods

### Plant materials

Annual tea cuttings (C. *sinensis* cv. Huangjinya) were bought from a tea plantation located at the tea plantation base of the Chaxi Valley Co., Ltd. in Tai’an, Shandong Province (36.19°N, 117.11°E). Here, the planting area is 200 m above sea level, the soil fertility is moderate, and tea plants grow well. In October 2019, we bought and transplanted the cuttings to a natural light greenhouse at Shandong Agricultural University and carried out a one-week-long seedling treatment under standard horticultural conditions.

After the one-week treatment, we selected at least 30 tea plants (C. *sinensis* cv. Huangjinya) and collected their flower buds (FBs), stems, terminal buds (TBs), first leaves under new shoots (FLs), second leaves under new shoots (SLs), third leaves under new shoots (TLs), and fourth leaves under new shoots (mature leaves, MLs). The harvested samples were immediately snap frozen in liquid nitrogen and stored at −80 °C for later use.

We also selected another 30 tea plants after the one-week-long seedling treatment, cleaned the soil from the roots, and fixed each plant in a hydroponic box with a foam board. Plants were fully aerated daily with an oxygen pump, and Hoagland’s nutrient solution (Xia, 2010) (Table S1) was replaced every 3.5 days. After one week of pre-culture, a PEG-induced, simulated drought treatment was initiated. PEG6000 concentrations ranged from 10% (mild drought stress) to 30% (moderate drought stress) to 50% (severe drought stress). At 2, 4 and 6 h after treatment, 2–3 mature leaves were removed at the same height from at least three tea seedlings and stored at −80 °C for later use. Mature leaves from plants that had not been subjected to drought treatment (PEG6000 treatment for 0 h) were used as the control; all other processing conditions were the same as in the PEG6000 treatment. Leaf samples were quickly frozen in liquid nitrogen and stored in a −80 °C freezer for later use.

### RNA extraction and quantitative real-time PCR analysis of the *Dof* genes

Total RNA was extracted with the RNAprep Pure Polysaccharide Polyphenol Plant Total RNA Extraction Kit (Tiangen, Cat No. DP441), and first-strand cDNA was synthesized using the Evo M-MLV RT Kit with gDNA Clean for qPCR (Accurate Biotechnology (Hunan) Co., Ltd, China) according to the manufacturer’s instructions. Real-time quantitative reverse transcription PCR (qRT-PCR) was used to detect the expression level of each gene using a cDNA template. Sixteen quantitative primers were designed using BD software (Tables S2). The internal reference was glyceraldehyde 3-phosphate dehydrogenase (GAPDH), which was synthesized by Sangon Biotech (Shanghai) Co., Ltd. GAPDH is considered to be the best reference gene under drought stress (Hao, 2012; Fang et al., 2017), and its expression does not differ among different developmental stages. We selected 16 *Dof* genes for qRT-PCR analysis (Table S3 and S4) using the SYBR^®^ Green Premix Pro Taq HS qPCR Kit (Accurate Biotechnology (Hunan) Co., Ltd, China). The 20 µL qRT-PCR reaction system contained 10.0 µL 2 ×ChamQ Universal SYBR qPCR Master Mix, 0.4 µL (10 µmol L^−1^) upstream and downstream primers, 1.0 µL template, and 8.2 µL ddH_2_O. Three technical replicates were performed for each sample. The reaction conditions were pre-denaturation at 95 °C for 30 s, followed by 40 cycles of 95 °C for 5 s and 60 °C for 30 s. A dissociation curve was drawn using 95 °C for 15 s, 60 °C for 60 s, and 95 °C for 15 s. The experimental data were quantitatively analyzed using the 2^−ΔΔCT^ method (Chen et al., 2017). We measured the expression of *Dof* genes in various tissues and in mature leaves under different simulated drought conditions. Each reaction was repeated three times, and the results are an average of three independent biological replicates.

### Database searches and identification of Dof family members in tea

The tea genome was downloaded from the Tea Genome Database (http://itak.feilab.net/cgi-bin/itak/index.cgi) (Xia et al., 2017). To identify all *Dof* genes in tea, the Dof domain (Pfam PF02701) was obtained from Pfam (http://pfam.xfam.org) (Finn et al., 2015). To verify the authenticity of candidate sequences, an HMM (hidden Markov model) profile of the Dof domain (PF02701) was used as a query to identify *Dofs* using the HMMER3.0 program (http://hmmer.janelia.org) (Finn et al., 2015). SMART (http://smart.embl.de) (Letunic, Doerks & Bork, 2015) and ScanProsite (http://www.expasy.ch/tools/scanprosite/) (Castro et al., 2006) were used to examine the CsDof domains of the deduced amino acid sequences (Table S4). The isoelectric points, molecular weights, instability indices, aliphatic indices, and grand average of hydropathicity (GRAVY) scores of the proteins were predicted using the ExPASy Proteomics Server (http://expasy.org/). If more than one allele was present in the genome file, we selected the longest allele for analysis. The Dof transcription factor sequences of Arabidopsis (Table S5) were downloaded from the Plant Transcription Factor Database (http://planttfdb.cbi.edu.cn/), and redundant genes were removed (Jin et al., 2014a). The CsDof proteins were used as BLASTP query sequences against the *Arabidopsis thaliana* (TAIR10) protein sequence file with default parameters (*E*-value <  1e^−5^) (Wang et al., 2019). Homologous Arabidopsis Dof proteins with the highest bit scores were used to construct a protein-protein interaction (PPI) network with STRING (version 10.0) (http://string-db.org/) (Damian et al., 2015), using the Arabidopsis database as the selected organism.

### Phylogenetic analysis, gene structure and motif identification

The Dof family protein sequences of Arabidopsis and tea were obtained as described above. All sequences were aligned using the default settings of ClustalX 2.1 (Larkin et al., 2007), and a phylogenetic tree was constructed with the neighbor-joining algorithm in MEGA 6.0 (http://www.megasoftware.net/mega6/). The reliability of the resulting tree was assessed using 1,000 bootstrap replicates. The structures of the *Dof* genes were analyzed online using the Gene Structure Display Server (GSDS) (http://gsds.cbi.pku.edu.cn/). The Dof family transcription factor database was downloaded from the Plant Transcription Factor Database (http://planttfdb.cbi.pku.edu.cn/) (Jin et al., 2014a). MEME Suite was used to identify motifs in the CsDof protein sequences (Bailey et al., 2009) using a motif width of 6–50 and a maximum of 15 motifs (Ma et al., 2015).

## Results

### Identification of the *CsDof* genes

We identified 16 non-redundant putative *CsDof* genes in the tea genome (Tables S3 and S4). Their lengths and predicted molecular weights varied widely, but there were fewer differences in their theoretical isoelectric points (Table 1). The predicted Dof transcription factors were 486 to 1,428 amino acids in length; their molecular weights ranged from 39.3 to 120.2 kDa, and their theoretical isoelectric points were close to 5 (4.99 to 5.22).

**Table 1:** Physiochemical properties of the tea *CsDof* genes and their corresponding proteins.

	Gene name	Gene ID	Size (aa)	Molecular weight (kDa)	PI	Instability index	Aliphatic index	Grand average of hydropathicity (GRAVY)
A	*CsDof1*	*CsA005492*	864	71.81608	5.05	60.65 unstable	25.93	0.948
B 1	*CsDof2*	*CsA012146*	1,047	87.01927	5.03	51.35 unstable	24.45	0.813
	*CsDof3*	*CsA012347*	870	72.83445	5.07	59.00 unstable	27.82	0.870
B 2	*CsDof4*	*CsA009371*	1,428	120.21870	5.00	44.41 unstable	26.47	0.745
	*CsDof5*	*CsA032220*	1,122	95.09702	5.04	45.53 unstable	28.34	0.804
C 1	*CsDof6*	*CsA002607*	1,047	86.47761	5.07	49.66 unstable	29.23	0.779
	*CsDof7*	*CsA002685*	966	79.51451	5.09	49.79 unstable	26.71	0.717
C 2.1	*CsDof8*	*CsA020146*	804	65.50705	5.13	51.25 unstable	30.35	0.794
	*CsDof9*	*CsA028787*	807	66.88200	5.12	51.32 unstable	30.98	0.832
C 2.2	*CsDof10*	*CsA027884*	744	61.30654	5.13	33.36 stable	26.61	0.756
C 3	*CsDof11*	*CsA002683*	765	64.08197	5.09	60.62 unstable	28.76	0.940
	*CsDof12*	*CsA007538*	930	78.49438	5.02	63.63 unstable	27.31	1.022
D 1	*CsDof13*	*CsA013235*	486	39.32758	5.22	32.61 stable	26.75	0.700
	*CsDof14*	*CsA013544*	1,407	116.56649	4.99	46.46 unstable	31.56	0.896
	*CsDof15*	*CsA021984*	1,389	113.63680	5.00	45.31 unstable	31.61	0.884
	*CsDof16*	*CsA027886*	1,311	107.98698	5.00	46.87 unstable	30.28	0.900

The instability index, the aliphatic index, and the GRAVY score were similar within each subfamily, but there were large differences among different subfamilies (Table 1). For example, the two members of the C2.1 subfamily had instability indices of 51.25 and 51.32 (unstable), aliphatic indices of 30.35 and 30.98, and GRAVY scores of 0.794 and 0.832. However, the two members of the C3 subfamily had instability indices of 60.62 and 63.63 (unstable), aliphatic indices of 28.76 and 27.31, and GRAVY scores of 0.94 and 1.022.

### Phylogenetic analysis and classification of *Dof* genes in tea and Arabidopsis

**Figure 1:**
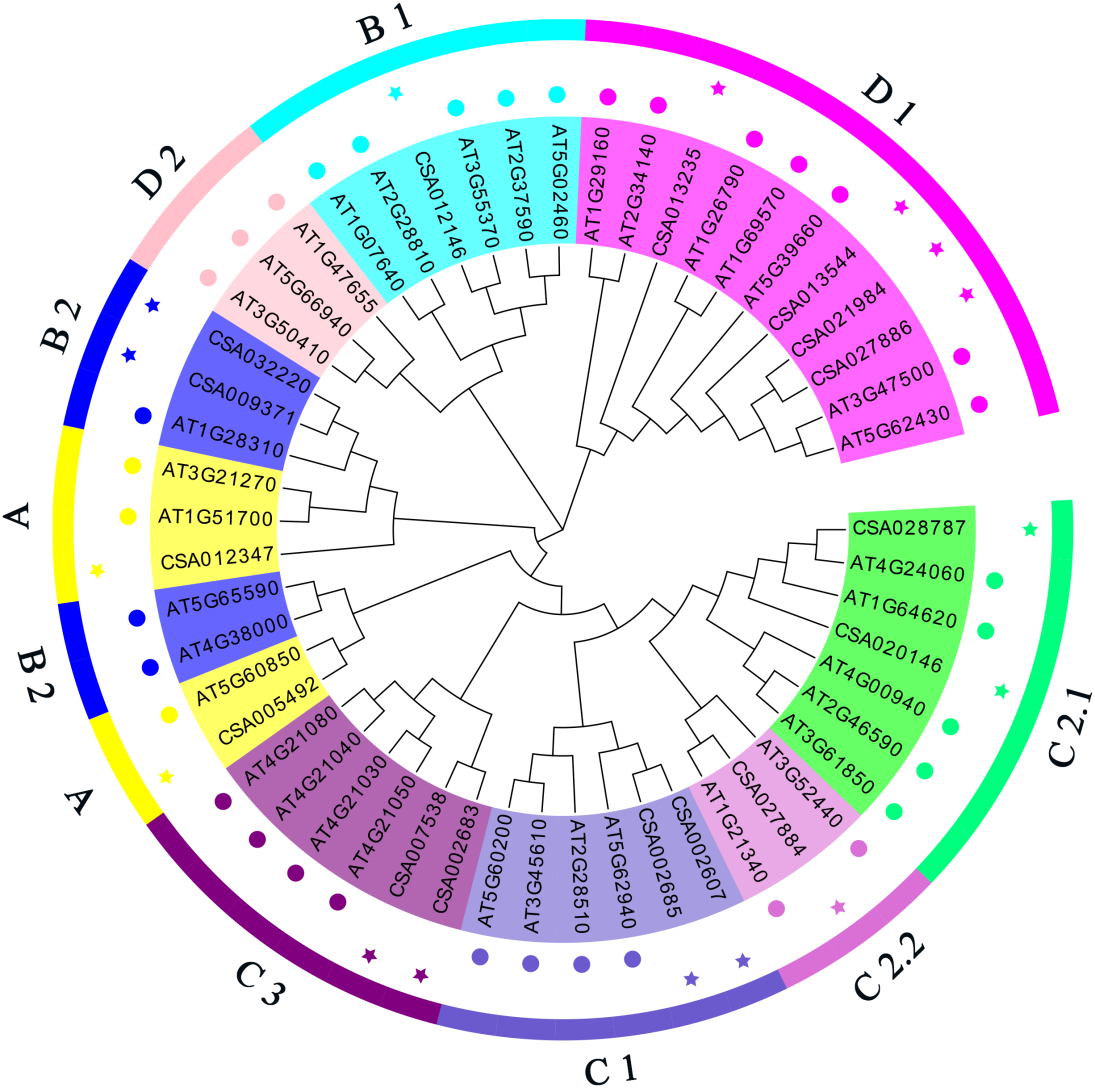
Phylogenetic relationships among *C. sinensis* and *A. thaliana* Dof proteins. The neighbor-joining tree was created using the MEGA6.0 program (bootstrap value set at 1,000). Thirty-six AtDof proteins marked with various colors pentacle and 19 CsDof proteins marked with various colors pentacle. The resulting phylogenetic tree was clustered into four major groups (A, B, C and D). The different colors of the pentacles represent different subfamilies.

To study the molecular evolution of the tea *CsDof* genes and predict their functions, tea and Arabidopsis Dof proteins (Tables S4 and S5) were used to construct a phylogenetic tree (Fig. 1). Based on the phylogenetic tree and previous reports, the predicted tea *CsDof* genes were divided into four major families (A, B, C, and D) and seven subfamilies (A, B1, B2, C1, C2, D1, and D2). Together, the C and D families constituted the largest group, including 11 members and accounting for 68.75% of the total number of predicted genes. Family B contained four members, accounting for 25% of the predicted genes, and Family A contained only one member, accounting for 6.25% of the predicted genes. By comparing tea and Arabidopsis Dof proteins, we found that Dof transcription factors from different species within the same family (any of the four major families A, B, C, and D) were more similar to one another than were Dof transcription factors from the same species in different families. For example, Arabidopsis and tea *Dof* genes classified into Family A were more similar to one another than were tea *Dof* genes classified into Families A and B.

### Gene structures and protein motifs of the *CsDof* gene family

**Figure 2:**
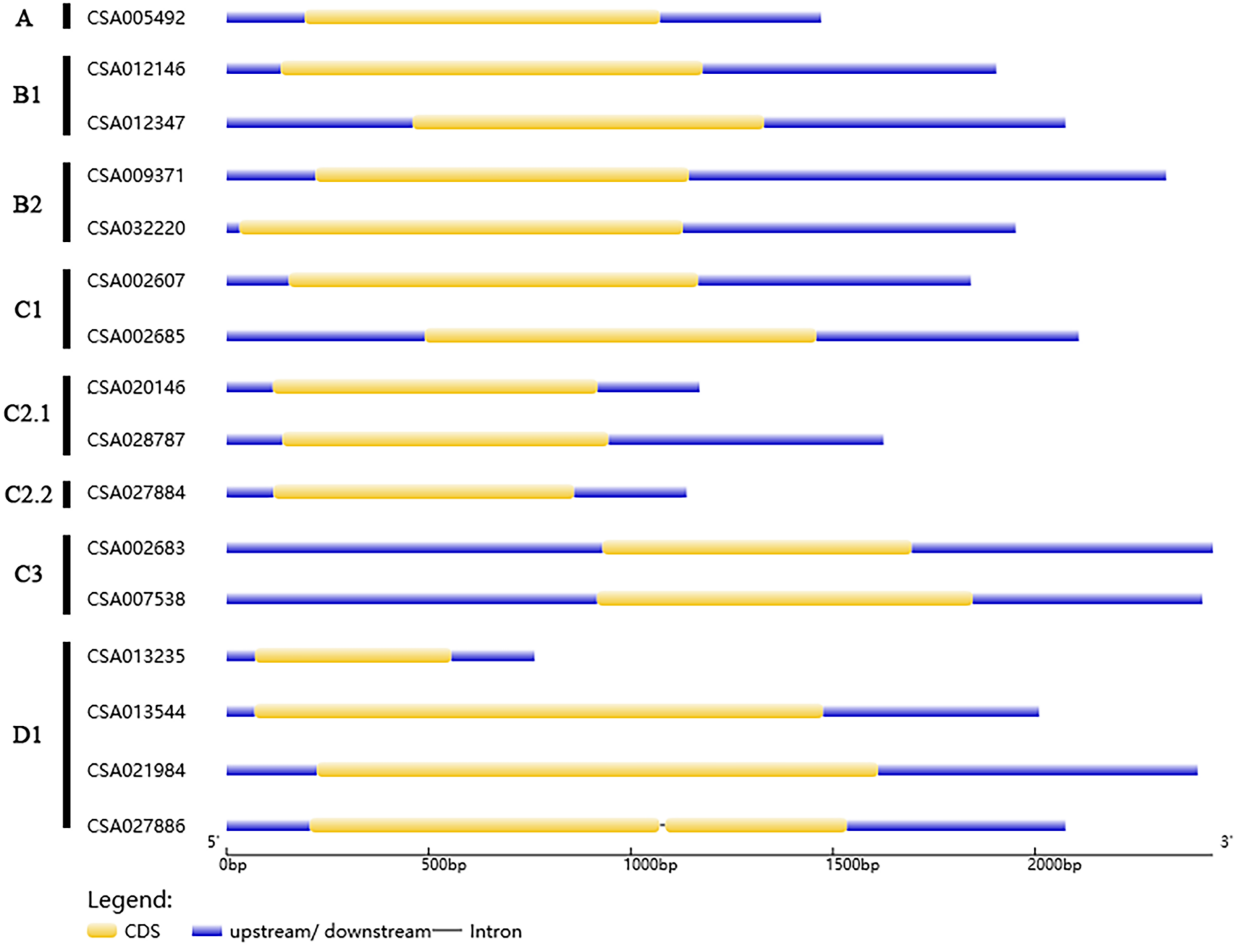
Phylogenetic analysis and structure of *Dof* genes in tea. In the gene structure diagram, yellow box, blue and black lines represent exons, upstream/downstream regions of the gene and introns, respectively.

**Figure 3:**
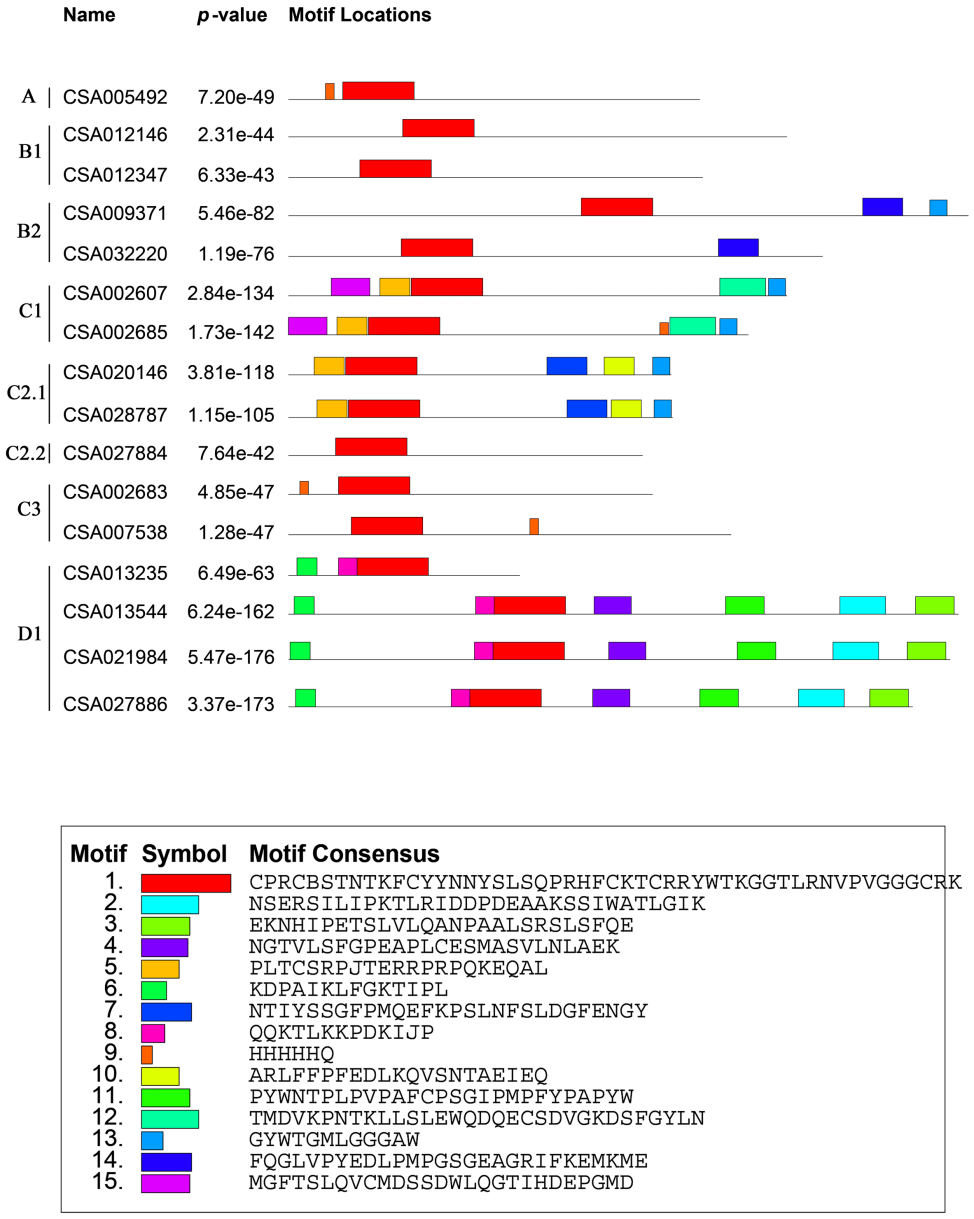
Common motifs of CsDof family proteins. Dof domains are represented by boxes of different colours.

**Figure 4:**
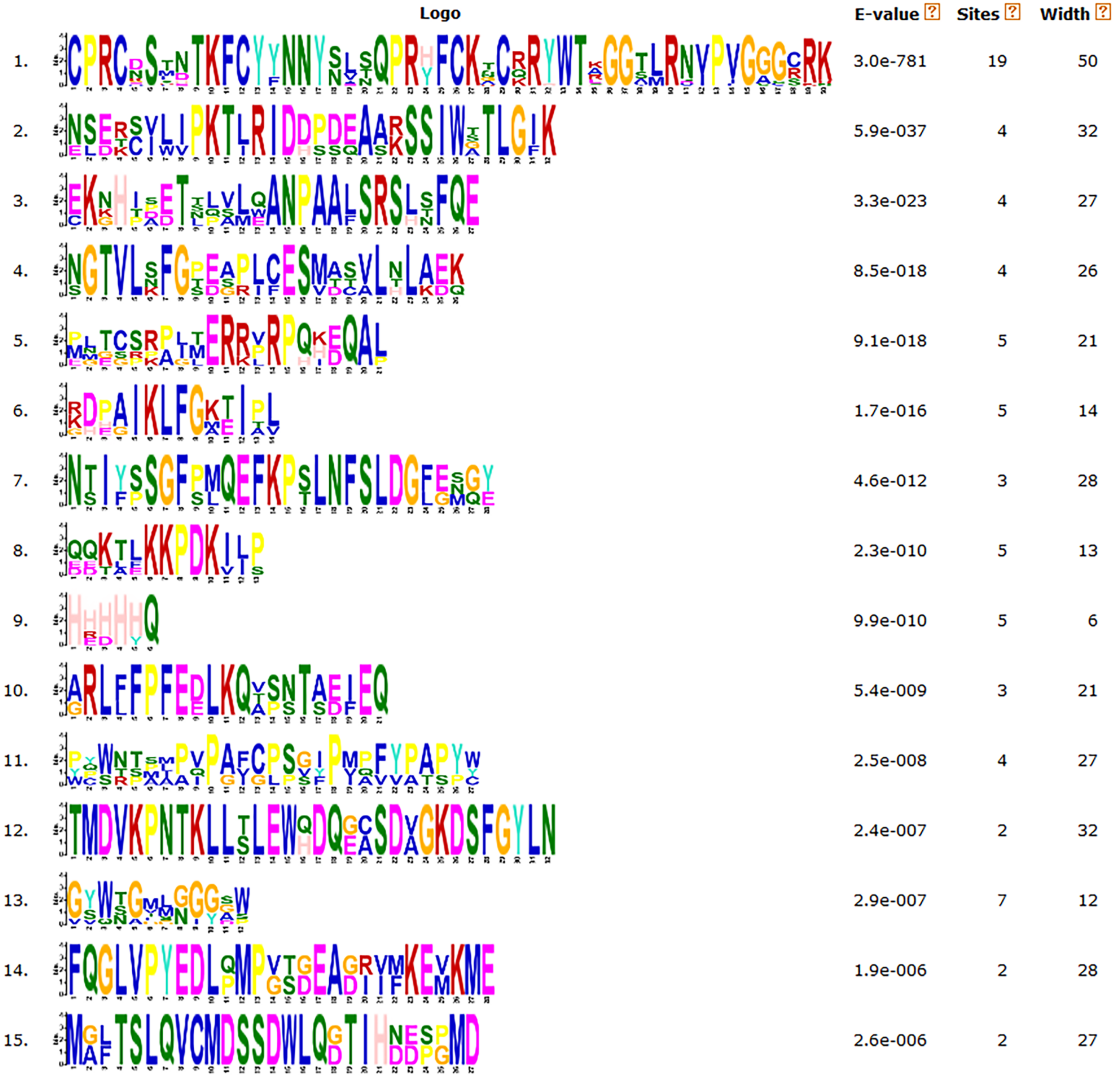
Sequence logos of tea Dof domains.

We analyzed the structures of the *CsDof* genes based on their coding sequences and genomic sequences (Fig. 2). The number of introns per gene ranged from zero to one. Only one *CsDof* gene (*CsDof16*) had an intron, and the others had no introns. In general, *CsDof* genes from the same subfamily had the same gene structure, indicating that tea *Dof* gene evolution is conserved. To reveal the diversity of Dof proteins in tea, we used the MEME Suite to identify motifs in the CsDof protein sequences. A total of 15 motifs were identified in the predicted CsDof proteins (Figs. 3 and 4). All CsDof proteins contained motif 1, which represents the conserved Dof domain. Furthermore, motif 13 was another conserved motif found in seven CsDofs. Basically, each subfamily had a specific motif, such as motif 14 in subfamily B2, motif 12 in subfamily C1, and motifs 7 and 10 in subfamily C2.1. Motifs 2, 3, and 11 were found only in subfamily D1. Several closely related CsDofs in the phylogenetic tree contained common motifs, suggesting that CsDofs from the same subfamily have similar functions. Analysis of gene structure and protein motif locations in the CsDofs indicated that most members were conserved in individual subfamilies.

### The Interaction Network of *Dof* TFs between *C. sinensis* and *Arabidopsis*

**Figure 5:**
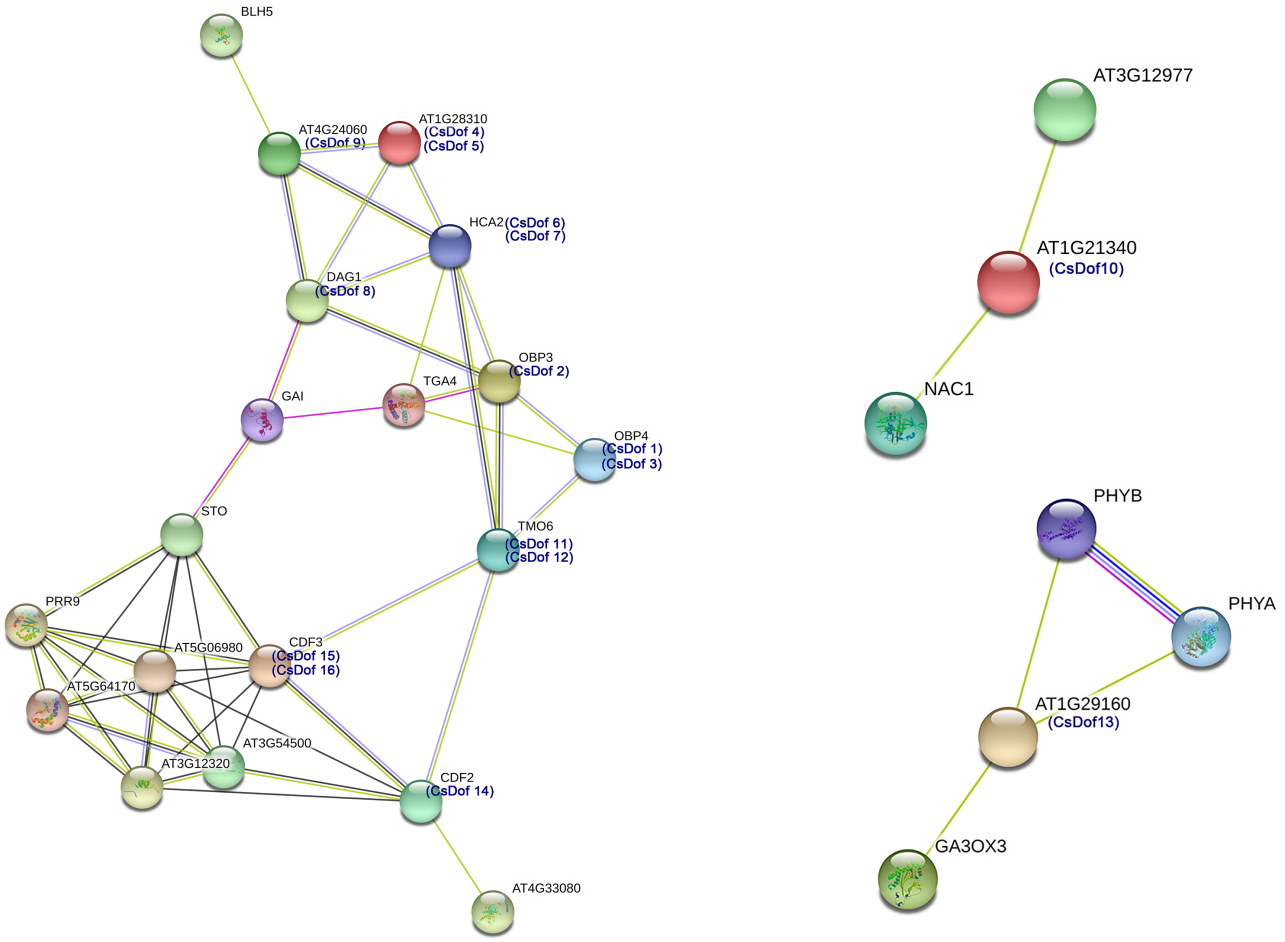
The interaction networks of Dofs in *C. sinensis* according to the orthologs in *Arabidopsis*.

We predicted protein-protein interactions between Arabidopsis homologs of the *CsDof* TFs and other Arabidopsis proteins (Fig. 5). Different line colors represent the types of evidence for the association. The amino acid sequence of CDF2 was highly similar to that of CsDof14, and the sequence of CDF3 was highly similar to those of CsDof15 and CsDof16. There was a predicted interaction between AT1G21340, which is highly similar to CsDof10, and the drought resistance protein NAC1 (Li et al., 2014). Moreover, AT1G29160, which was highly similar to CsDof13, was predicted to have complex interactions with the drought resistance-related protein PHYB (Yoo et al., 2017) and the abiotic stress protein GA3ox3 (Pan et al., 2017). In addition, HCA2, which was highly similar to CsDof6 and CsDof7, was predicted to have complex interactions with seven CsDofs (CsDof2, CsDof4, CsDof5, CsDof8, CsDof9, CsDof11 and CsDof12). Similarly, OBP3, which was highly similar to CsDof2, was also predicted to have complex interactions with seven CsDofs (CsDof1, CsDof3, CsDof6, CsDof7, CsDof8, CsDof11 and CsDof12).

### Tissue expression patterns of CsDof genes

**Figure 6:**
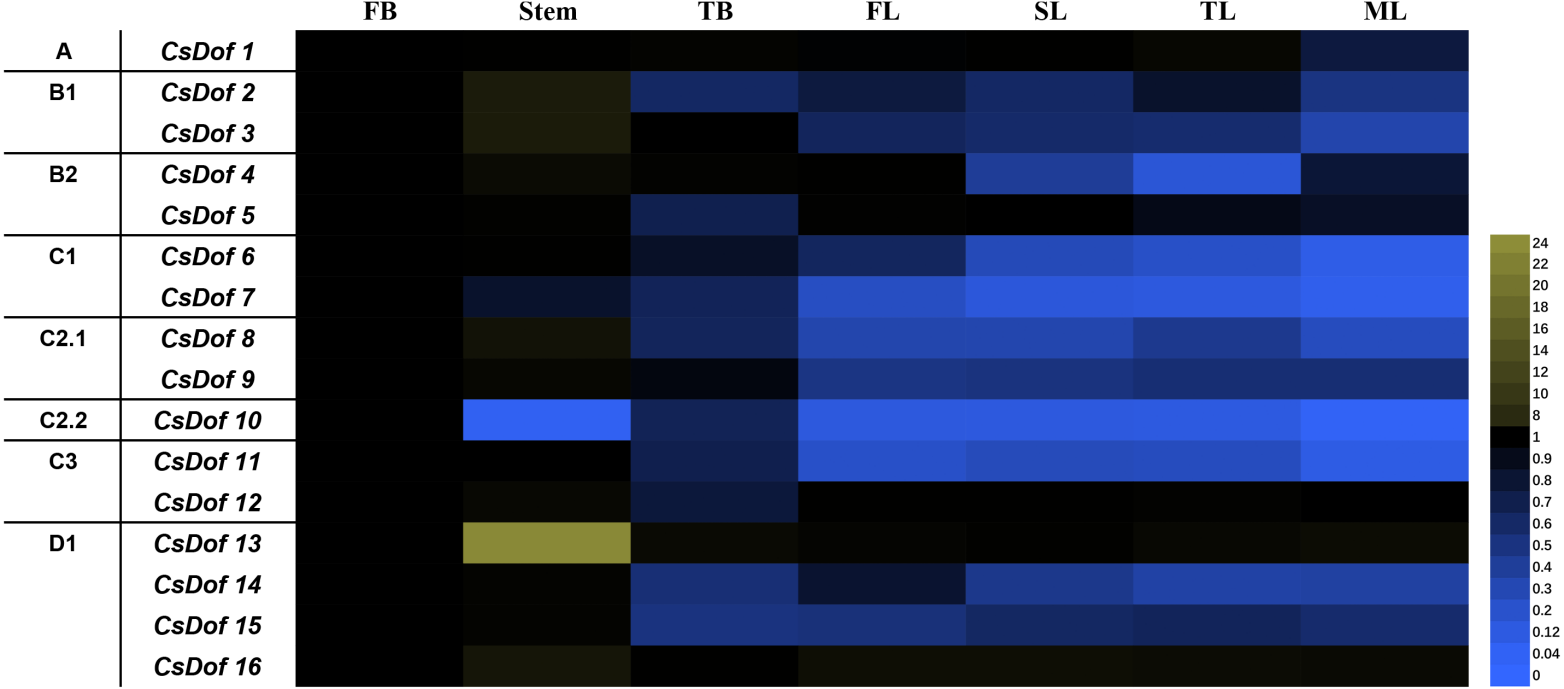
Relative expression profiles of *Dof* genes in different tissues of tea plants. The gene expression of different tissues of tea plants was analyzed by qRT-PCR. Expression levels were normalized against that of GAPDH. FB denotes the flower bud, TB means the terminal bud, FL denotes the first leaf of new sprouting shoots, SL means the second leaf, TL denotes the third leaf and ML means the mature fourth leaf.

Sixteen *CsDof* genes were expressed in the flower buds (FBs), stems, terminal buds (TBs), first leaves under new shoots (FLs), second leaves under new shoots (SLs), third leaves under new shoots (TLs), and fourth leaves under new shoots (mature leaves, MLs). There were differences in expression patterns among different tissues (Fig. 6). For example, the expression levels of *CsDof7* and *CsDof10* were higher in FBs than in other tissues, and *CsDof2*, *CsDof3*, *CsDof8*, *CsDof13*, and *CsDof16* had the highest expression in stems. Approximately 56.25% of the *CsDof* genes were significantly downregulated in TBs compared to FBs. The expression levels of *CsDof4* and *CsDof14* in TLs were significantly lower than those in other tissues. *CsDof* 8 and *CsDof9* were significantly downregulated in FLs. Eleven (68.75%) of the *CsDof* genes were significantly downregulated in SLs compared to FBs*. CsDof1*, *CsDof2*, *CsDof3*, and *CsDof6* were significantly downregulated in MLs. The expression levels of *CsDof16* were similar among all tissues.

### Expression patterns of *CsDof* genes under PEG6000-induced drought stress

**Figure 7:**
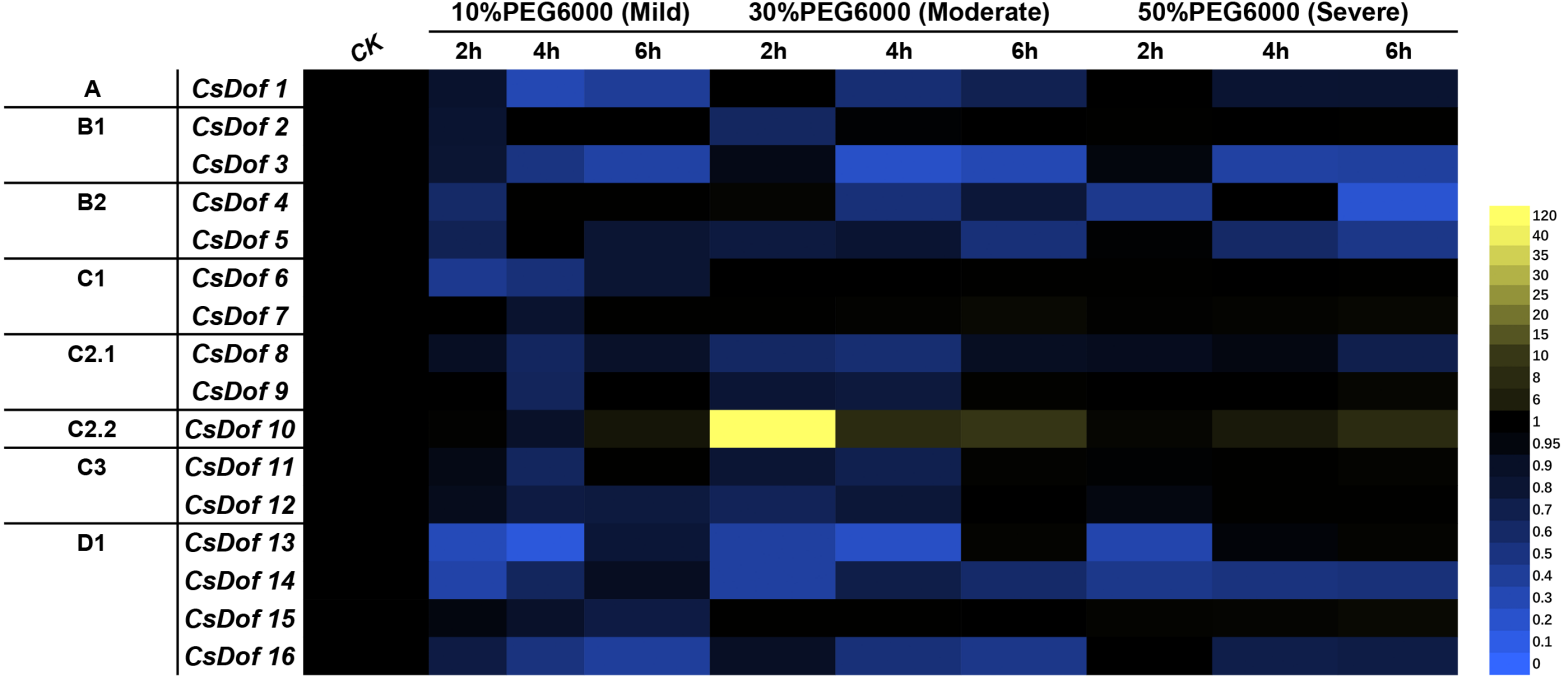
Expression patterns of *CsDof* genes in response to drought stress in tea plant cultivar ‘Huangjinya’. Mild means mild drought stress, Moderate means moderate drought stress, Severe means severe drought stress.

We analyzed the expression of 16 *CsDof* genes at 0, 2, 4 and 6 h after exposure to different degrees of PEG6000-induced drought stress (mild, moderate and severe drought stress) (Fig. 7) and found that most *CsDof* genes responded to drought stress.

The expression of *CsDof10* after 6 h of mild drought stress was significantly higher than its expression under the control treatment. *CsDof1*, *CsDof3*, *CsDof6*, *CsDof8*, *CsDof12* and four genes in the D1 subfamily were significantly downregulated after 2, 4 and 6 h of mild drought stress. *CsDof2* and *CsDof4* were significantly downregulated only after 2 h of mild drought stress. The genes whose expression levels decreased significantly after 4 h of mild drought stress were *CsDof7*, *CsDof9* and *CsDof10*.

*CsDof10* was significantly upregulated after 2 h of moderate drought stress; its expression declined gradually at 4 and 6 h but remained significantly higher than that of control plants. In addition, 75% (12) of the *CsDof* genes showed significantly lower expression under moderate drought stress than under control conditions, and the expression of *CsDof5*, *CsDof8*, *CsDof14* and *CsDof16* continued to decrease after 2, 4, and 6 h of drought treatment.

Under severe drought stress, the expression of 43.75% (7) genes was significantly lower than under the control treatment, and the expression of *CsDof1*, *CsDof3*, *CsDof5*, *CsDof14*, and *CsDof16* continued to decrease after 2–6 h of treatment. *CsDof13* was significantly downregulated only when treated with severe drought stress for 2 h. Interestingly, the expression of *CsDof10* showed a gradual increase with time, and its expression was the highest after 6 h of severe drought stress.

## Discussion

Li et al. (2016) identified 29 tea tree Dof genes and predicted that *CsDof-22* interacted with ABA1 and participated in drought stress. However, Li et al. (2016) only studied the expression of 8 of the 29 *Dof* family members at different time points under single levels of high temperature, low temperature, drought stress, and salt stress. They found that only *CsDof-8* and *CsDof-13* responded to drought stress at the transcriptional level and that *CsDof-22* did not change significantly at the transcriptional level compared to the control. Here, we identified 16 new members of the *Dof* gene family in tea and focused on their role in the mechanism of drought response under different degrees of drought stress. We specifically studied the response of the 16 new members to light, moderate and severe drought stress over time, expanding our understanding of the role of tea tree *Dofs* in the response to drought stress.

### Dof gene numbers in multiple plant species

With advances in genome sequencing technology, members of the *Dof* gene family have been identified in many species, including Arabidopsis (Kushwaha et al., 2011), tomato (Cai et al., 2013), rice (Lijavetzky, Carbonero & Vicente-Carbajosa, 2003), castor bean (Jin, Chandrasekaran & Liu, 2014b), peach (Chen et al., 2017), eggplant (Wei et al., 2018b), physic nut (Zou & Zhang, 2019), and others. The tea genome has been sequenced (Wei et al., 2018a; Xia et al., 2017), and Li et al. (2016) identified 29 putative Dof TFs. In this work we identified 16 new *CsDof* genes (Fig. S2).

**Figure 8:**
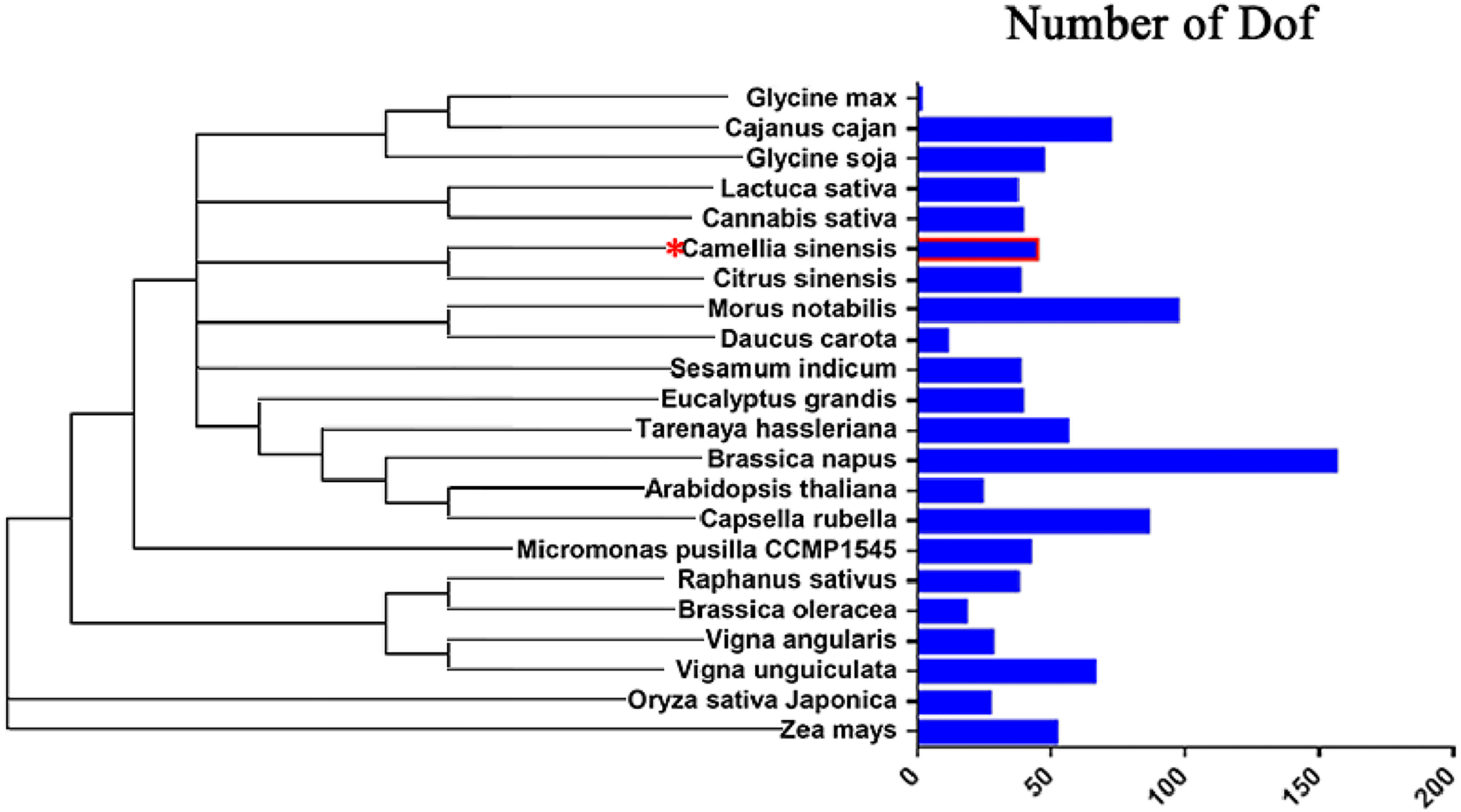
Distribution of Dof transcription factors in different species.

To study the evolution of *Dof* genes in plants, we compared 22 different algal and plant species, including species from the Chlorophyta and the Embryophyta subkingdoms, and determined how many *Dof* genes were present in each species (Chen et al., 2017; Letunic & Bork, 2007; Letunic & Bork, 2011). The number of *Dof* genes in different species ranged from 1 to 156. Embryophyte species had more *Dof* genes than chlorophyte algae (Fig. 8), suggesting that *Dof* genes have played an important role in the evolutionary process.

### Duplication of the *CsDof* genes

Dof transcription factors are found not only in angiosperms and gymnosperms but across all plant lineages, presumably because a longer breeding time has led to a greater diversity of the Dof family and the Dof transcription factors participate in more biological processes (Yanagisawa, 2004; Yin et al., 2017). The Dof family has been found in Arabidopsis (Kushwaha et al., 2011), tomato (Cai et al., 2013), rice (Lijavetzky, Carbonero & Vicente-Carbajosa, 2003) and peach (Chen et al., 2017), which have 36, 34, 30, and 25 Dof transcription factors, respectively. To date, a total of 45 *CsDof* genes have been reported in tea (Li et al., 2016), but the number of *Dof* family members is independent of genome size. For example, the number of *Dof* family members in peach (25) (Chen et al., 2017) is less than that in Arabidopsis (36) (Kushwaha et al., 2011), although the genome size of peach (224.6 Mb) is almost twice that of Arabidopsis (125 Mb). We found that the number of Arabidopsis *Dofs* was four-fifths that of tea (45), despite the fact that the tea genome (3.1 Gb) is 25 times bigger than that of Arabidopsis (Xia et al., 2017). Studies have shown that more than half of the bases (67%) in the tea genome are retrotransposon sequences, with numerous copies and insertions into different sites, leading to a dramatic expansion of genome size (Xia et al., 2017). Therefore, although the size of the tea genome is much larger than that of Arabidopsis, the number of Dof family members is only slightly greater, perhaps due to gene duplication during evolution.

### Homology analysis of the tea and Arabidopsis Dof genes

Dof proteins in Arabidopsis are usually divided into four families (A–D) (Kushwaha et al., 2011). We also divided the tea *Dof* genes into four families based on the positions of their proteins on a phylogenetic tree. Moreover, we found that gene structures were consistent within families, suggesting that the genes within a family may have similar functions (Chen et al., 2017).

Papi et al. (2000) demonstrated that AtDAG1 (Dof affecting germination) is expressed in flowers and mature pericarp tissue, mainly in the seed coat and phloem. We found that tea genes assigned to the C2.1 subfamily with *AtDAG1* were mainly expressed in flower buds, stems, and terminal buds. Their expression levels in leaves were lower, consistent with previous studies (Papi et al., 2000). Therefore, we speculate that genes of the C2.1 subfamily in tea are similar to those in Arabidopsis and that most of them are involved in the plant vascular system and seed development (Gabriele et al., 2009). The results of gene expression analysis provide a basis for the functional characterization of *CsDof* genes, and the phylogenetic analysis of the *Dof* family provides a theoretical basis for further functional genomics studies in tea.

### Transcript profiles of CsDof paralogs

We found that the expression patterns of the two C1 subfamily members differed: *CsDof7* (*CsA002685)* was mainly expressed in flower buds, whereas *CsDof6* (*CsA002607*) was primarily expressed in flower buds and stems. Some genes and their paralogs play redundant roles (Fornara et al., 2009), but other paralogs, such as *AtDof3.4* (*OBP1*) and *AtDof5.8* (*SCAP1*), have different functions. Although both are OG-2b orthologs (Zou & Zhang, 2019), *AtDof3.4* participates in defensive response (Zhang et al., 1995) and cell cycle regulation (Skirycz et al., 2008), whereas *AtDof5.8* (*SCAP1*) participates in vascular development (Konishi & Yanagisawa, 2007), stomatal function and morphogenesis (Negi et al., 2013). Therefore, we speculate that genes that are expressed differently in some subfamilies may be involved in different growth and developmental processes. Moreover, differences in the expression of *Dof* genes from the same subfamily may be related to sequences other than conserved motifs.

### CsDof proteins may interact with proteins that respond to drought stress

We predicted protein-protein interactions between Arabidopsis homologs of the *CsDof* TFs and other Arabidopsis proteins. In Arabidopsis and tomato, CDFs are involved in the response to drought stress (Corrales et al., 2014; Hoekstra, Golovina & Buitink, 2001; Rizhsky, 2004). We predicted by protein-protein interaction network analysis that the CDF2 amino acid sequence was highly similar to that of CsDof14, and the CDF3 sequence was highly similar to those of CsDof15 and CsDof16. Moreover, CDF3 was predicted to have complex interactions with CDF2, STO (salt tolerance protein), CsDof11, CsDof12, and five other proteins (AT5G06980, PRR9, AT5G64170, AT3G54500, AT3G12320). Similarly, CsDof10 was predicted to interact with NAC1 (drought resistance protein) (Li et al., 2014), and CsDof13 was predicted to interact with PHYB, which is involved in drought resistance (Yoo et al., 2017). Therefore, we speculate that CsDof10, CsDof13, CsDof14, CsDof15, CsDof16 may play important roles in the drought stress response. Moreover, CsDof15 and CsDof16 may participate in the drought stress response through interaction with CsDof11, CsDof12 and CsDof14.

### The Dof gene family may be involved in drought stress response

In this study, we investigated the responses of *CsDof* genes to varying degrees of PEG6000-induced drought stress. Most *CsDofs* responded to different degrees of drought stress, although the details of their responses differed. This suggests that the *CsDof* genes may play various roles in drought stress.

Corrales et al. (2014) found that all *SlCDF* genes, which are members of the *Dof* gene family in tomato, are regulated by drought and that members of this gene family may be upstream activators of drought stress response pathways, directly or indirectly acting on different stress-regulated target genes (Corrales et al., 2014). In Arabidopsis, the overexpression of *SlCDF3* promoted the accumulation of compounds such as proline, glutamine, GABA and sucrose (Hoekstra, Golovina & Buitink, 2001; Rizhsky, 2004). The levels of these compounds usually change significantly under drought stress (Kerepesi & Galiba, 2000; Farrant & Moore, 2011; Pinheiro & Chaves, 2011), increasing stress tolerance through osmotic adjustment, detoxification of ROS, and intracellular pH regulation (Munns & Tester, 2008; Bressan, Bohnert & Zhu, 2009; Chaves, Flexas & Pinheiro, 2008), Here, we found that *CsDof15* (*CsA021984*) and *CsDof16* (*CsA027886*) exhibited up to 100% identity with *SlCDF1* and *SlCDF3* (Fig. S1) and were responsive to drought stress. In particular, the expression of *CsDof16* under various levels of drought stress gradually decreased through time. By contrast, *CsDof15* expression showed a gradual downward trend only under mild drought stress as the treatment time increased from 2 to 6 h. We found only one C2.2 subfamily member (*CsDof10*) whose expression was significantly upregulated after 2 h of moderate drought stress in comparison to the control condition. Thus, *CsDof10* may play an important role in moderate drought stress.

## Conclusions

In summary, 16 new *CsDof* genes were identified. Analysis of their physiochemical properties, phylogeny, gene structure and PPI network provided more complete information for this gene family in tea. Gene expression profiles after drought stress indicated that some of the *CsDof* s may play a role in drought resistance. The results of this study provide a basis for future functional characterization of the role of *Dof* genes in drought stress in eukaryotes.

##  Supplemental Information

10.7717/peerj.9269/supp-1 Supplemental Information 1 Supplemental Files

## Additional Information and Declarations

### DNA Deposition

The following information was supplied regarding the deposition of DNA sequences: The 16 CsDofs sequences listed in Table 1 are available at http://itak.feilab.net/cgi-bin/itak/db_family_gene_list.cgi?acc=C2C2-Dof&plant=4442.

### Data Availability

The following information was supplied regarding data availability: The raw gene data of tea and Arabidopsis are available in Tables S4 and S5. The alignment analysis of the amino acid sequences of Dof (CsDof1-29 amino acid sequence was already studied (Li et al., 2016) and 16 distinct CsDofs we identified) is available in Fig. S2.
